# Inhibition of lncRNA-NEAT1 sensitizes 5-Fu resistant cervical cancer cells through de-repressing the microRNA-34a/LDHA axis

**DOI:** 10.1042/BSR20200533

**Published:** 2021-07-22

**Authors:** Xuecheng Shao, Xuehui Zheng, Dan Ma, Yang Liu, Guoyan Liu

**Affiliations:** 1 Department of Obstetrics and Gynecology, The Third Central Clinical College of Tianjin Medical University, Tianjin 300170, China; 2 Department of Obstetrics and Gynecology, Characteristic Medical Center of Chinese People's Armed Police Force, Tianjin 300170, China; 3 Department of Obstetrics and Gynecology, Tianjin Third Central Hospital, Tianjin 300170, China; 4 Department of Obstetrics and Gynecology, Tianjin Medical University General Hospital, Tianjin 300170, China

**Keywords:** 5-Fu resistance, cervical cancer, lncRNA-NEAT1, miR–34a, Warburg effect

## Abstract

Cervical cancer is one of the most diagnosed malignancies among females. The 5-fluorouracil (5-Fu) is a widely used chemotherapeutic agent against diverse cancers. Despite the initially encouraging progresses, a fraction of cervical cancer patients developed 5-Fu resistance. We detected that nuclear-rich transcripts 1 (NEAT1) was significantly up-regulated in cervical cancer tissues and cell lines. Moreover, NEAT1 was positively associated with 5-Fu resistance. Furthermore, expression of NEAT1 was significantly up-regulated in 5-Fu resistant CaSki cervical cancer cells. Knocking down NEAT1 by shRNA dramatically promoted the sensitivity of 5-Fu resistant CaSki cells. We observed a negative correlation between long noncoding RNA (lncRNA)-NEAT1 and miR-34a in cervical cancer patient tissues. Overexpression of miR-34a significantly sensitized 5-Fu resistant cells. Bioinformatics analysis uncovered that NEAT1 functions as a competitive endogenous RNA (ceRNA) of miR-34a in cervical cancer cells via sponging it at multiple sites to suppress expression of miR-34a. This negative association between NEAT1 and miR-34a was further verified in cervical cancer tissues. We found the 5-Fu resistant cells displayed significantly increased glycolysis rate. Overexpression of miR-34a suppressed cellular glycolysis rate and sensitized 5-Fu resistant cells through direct targeting the 3′-untranslated region (UTR) of LDHA, a glycolysis key enzyme. Importantly, knocking down NEAT1 successfully down-regulated LDHA expressions and glycolysis rate of cervical cancer cells by up-regulating miR-34a, a process could be further rescued by miR-34a inhibition. Finally, we demonstrated inhibition of NEAT1 significantly sensitized cervical cancer cells to 5-Fu through the miR-34a/LDHA pathway. In summary, the present study suggests a new molecular mechanism for the NEAT1-mediated 5-Fu resistance via the miR-34a/LDHA-glycolysis axis.

## Introduction

Cervical cancer, one of the commonly diagnosed malignancies among females, is the main leading cause of female tumor deaths around the world [1]. The high risks of morbidity and mortality rate of cervical cancer seriously impaired the quality of lives since most cervical cancer patients are diagnosed in the advanced stage [1,2]. Although diagnosis and treatment of cervical cancer have been improved, the prognosis of patients still remains poor due to metastasis and chemoresistance [3,4]. 5-fluorouracil (5-Fu) is one of the systemic adjuvants and palliative chemotherapies against cervical cancers [5]. Recently, multiple strategies including the 5-Fu-based combination with other chemotherapeutic agents have been developed to enhance the anti-tumor activity of 5-Fu [6]. Despite the initially encouraging progresses in cervical cancer therapy, a fraction of patients’ response to 5-Fu gradually attenuated, resulting from the development of chemoresistance [7]. Consequently, 5-Fu resistance renders it a major challenge for the 5-Fu-based chemotherapy. Thus, investigating the underlying molecular mechanisms of chemoresistance is an essential task for developing novel therapeutic strategies against cervical cancer.

Long noncoding RNAs (lncRNAs), which consist of approx. 200 nucleotides, is a class of long-non-coding RNAs [8]. They are known to play critical roles in various biological cellular processes as well as malignant diseases via regulating target genes expressions [9]. Accumulating evidence have characterized lncRNAs to act as essential regulators during the initiation and development of cervical cancer [9]. Among them, the lncRNA CRNDE has been reported to promote the growth and metastasis of cervical cancer cells through suppressing PUMA [10]. LncRNA nuclear-rich transcripts 1 (NEAT1) has been found to play an oncogenic role in diverse tumors, such as endometrial cancer [11], breast cancer [12], lung cancer [13], colon cancer [14] and gastric cancer [15]. However, the precise roles of lncRNA NEAT1 in 5-Fu resistance and the underlining molecular mechanisms in cervical cancer has not been elucidated. As another class of short, noncoding RNA, microRNAs are capable to bind the 3′-untranslated region (UTR) of their target genes to suppress the corresponding mRNA translation and stability [16]. Similar to lncRNAs, miRNAs play crucial roles in various cancer processes [17]. Studies demonstrated that miR-34a participates in the initiation and development of various cancers, including cervical cancer [18]. However, the roles of the interaction of miR-34a with lncRNA NEAT1 in chemosensitivity of cervical cancer are unmasked.

Cancer cells adapt to tumor microenvironment by majorly depending on aerobic glycolysis for not only energy production but providing essential metabolic intermediates for biosynthesis of macromolecules, a phenomenon called the ‘Warburg effect’ [19]. Moreover, this new hallmark of cancer cells has been revealed to be associated with chemoresistance from preclinical and early clinical studies [20]. Therefore, effective targeting of the dysregulated glycolytic pathway has emerged as a therapeutic approach to overcome chemoresistance of cervical cancers.

In the present study, the precise roles of lncRNA-NEAT1 in 5-Fu resistance of cervical cancer will be investigated. We found overexpression of NEAT1 de-sensitized cervical cancer cells to 5-Fu treatment. Molecular mechanism studies revealed that NEAT1 functions as a competitive endogenous RNA (ceRNA) of miR-34a and positively regulates the expression of LDHA, a glycolysis speed limit enzyme. These results indicate effective targeting the NEAT-miR-34a-LDHA axis could contribute to developing new therapeutic agents against 5-Fu resistant cervical cancer.

## Materials and methods

### Tumor tissues collection and ethics statement

The present study was approved by the medical ethics committee of Tianjin Medical University General Hospital and complied with the Declaration of Helsinki. A total of 35 cervical cancer tissues and their matched adjacent normal tissues were obtained from patients who underwent surgery from January 2017 to June 2018 in the Department of Obstetrics and Gynecology, Tianjin Medical University General Hospital, China. All patients did not receive chemo- or radio-therapy before tissues collection. After surgical removal, tissue samples were frozen immediately in liquid nitrogen and stored at −80°C until use. The clinical histopathological diagnosis of cervical cancer tissues was approved by pathologists. All patients agreed to and gave written informed consent.

### Cell culture and reagents

Human normal cervical epithelial cell line, Ect1/E6E7 and cervical cancer cells Hela, C-33A, SiHa and CaSki were obtained from obtained from the Cell Bank of the Chinese Academy of Sciences (Shanghai, China). Cells were cultured in RPMI 1640 medium (Gibco, Carlsbad, CA, U.S.A.) supplemented with 10% fetal bovine serum (FBS) (Bio-Rad, Hercules, CA, U.S.A.) and 100 IU/ml penicillin G and 100 μg/ml streptomycin (Sigma–Aldrich, St. Louis, MO, U.S.A.) at 37°C in a 5% CO_2_ atmosphere. The establishment of 5-Fu resistant cervical cancer cell line was performed according to previous report [21]. Briefly, CaSki parental cells were exposed to gradually increasing 5-Fu concentrations from 5 to 20 μM for 4 months to select the survival cell clones, which were further combined and frozen for downstream experiments. Rabbit monoclonal anti-LDHA antibody (#3582) was purchased from Cell Signaling Technology Inc. (Danvers, MA, U.S.A.). Mouse monoclonal anti-β-actin (A2228) was purchased from Sigma–Aldrich (St. Louis, MO, U.S.A.). 5-Fu was purchased from Sigma–Aldrich (St. Louis, MO, U.S.A.).

### Plasmid, shRNA and miRNAs’ transfections

The transfections of plasmid DNA, shRNA and miRNAs were performed using the Lipofectamine 2000 (Thermo Fisher Scientific, Inc., Waltham, MA, U.S.A.) according to the manufacturer’s instructions. Briefly, 3 × 10^5^ cells were seeded in six-well plates and cultured overnight to reach 70% confluence. Then plasmid DNA, miRNA, or shRNA was diluted with 200 μl of OPTI-MEM (Invitrogen, Thermo Fisher Scientific Inc.). Meanwhile, 5 μl of Lipofectamine 2000 was added and the mixture was incubated at room temperature for 10 min. The mixture was added into cell culture medium for 48 h. Plasmid DNA was transfected at 1 μg; miRNAs were transfected at 100 nM; and shRNA was transfected at 500 nM. The control miRNA, miR-34a or anti-miR-34a precursor was purchased from RiboBio (Guangzhou, Guangdong, China). The oligonucleotides of the control shRNA (CGUACGCGGAAUACUUCGAUU) and shRNA NEAT1 (GUGAGAAGUUGCUUAGAAACUUU) were synthesized by GenePharma Co. (Shanghai, China). NEAT1 overexpression vector was cloned into the pcDNA3.1 plasmid vector according to previous descriptions [22]. The LDHA overexpression vector was purchased from Origene.com. Experiments were repeated three times.

### Predictions of miRNA–mRNA and miRNA–lncRNA binding

The prediction of lnRNA-NEAT1 and miR-34a interaction was performed by the starBase of ENCORI (http://starbase.sysu.edu.cn/). The sequence analysis for the binding sites of miR-34a on 3′UTR of LDHA was performed through TargetScan.org.

### Cell viability assay

Cell viability was examined by MTT (3-(4,5-dimethylthiazol-2-yl)-2,5-diphenyltetrazolium bromide) method (Sigma–Aldrich, Shanghai, China) according to the manufacturer’s instructions. Briefly, approximately 5 × 10^3^ cells were seeded in 96-well plates to reach 70% confluence. After treating with 5-Fu at the indicated concentrations, the medium was removed. After washing by PBS, 0.5 mg/ml, 20 μl MTT solution was added into each well and incubated at 37°C for 2 h. Cells were then incubated with 100 μl DMSO for 2 h at 37°C. The optical density (OD) value of formazan concentrations was determined at 540 nm. Results were normalized by cell numbers. All experiments were carried out in triplicate and repeated three times.

### Cell death assay

Cell death was examined by flowcytometry analysis using an Annexin V-FITC/PI apoptosis kit (Invitrogen) according to the manufacturer’s instructions. Cervical cancer cells were collected and washed with cold PBS. Cells were incubated with 5 μl FITC-Annexin V and 1 μl PI working solution (100 μg/ml) for 15 min with light protection. Intensity of fluorescence was examined by flow cytometry analysis (FACS Calibur™, BD Biosciences, CA, U.S.A.). Experiments were repeated three times.

### RNA extraction and qRT-PCR

Total RNA was isolated from cervical tissues and cells using the TRIzol (Invitrogen) method according to the manufacturer’s protocols. RNA samples were treated with DNase to eliminate genomic DNA. The quality and quantity of RNA were examined using ND-1000 spectrophotometer (NanoDrop Technologies Inc., Wilmington, U.S.A.). For mRNA and lncRNA detections, a total of 1 μg RNA was reversely transcribed into cDNA using a High-Capacity cDNA Reverse Transcription Kit (Applied Biosystems, Foster City, CA, U.S.A.). For miRNA expressions detection, a TaqMan miRNA Reverse Transcription Kit (Applied Biosystems) was applied to synthesize cDNA following the manufacturer’s protocol. The RT-PCRs were performed using the SYBR Green qPCR Master Mix (Thermo Fisher Scientific, Shanghai, China) by the ABI PRISM 7300 real-time PCR system (Applied Biosystems). β-actin was used as internal control to normalize the relative mRNA and lncRNA expression levels. Human U6 was used to normalize the relative miRNA expression levels. The thermal cycle was set as follows: 95°C for 1 min and 40 cycles at 95° C for 15 s, 58°C for 20 s and 72°C for 20 s. The primers used in the present study were synthesized by GenePharma Co. (Shanghai, China) as follows: NEAT1: Forward: 5′-ATGCCACAACGCAGATTGAT-3′ and Reverse: 5′-CGAGAAACGCACAAGAAGG-3′; HK2: Forward: 5′-TACACTCAATGACATCCGAACTG-3′ and Reverse: 5′-CGTCCTTATCGTCTTCAATATCC-3′; PKM2: Forward: 5′-TCGAGGAACTCCGCCGCCTG-3′ and Reverse: 5′-CCACGGCACCCACGGCGGCA-3′; LDHA: Forward: 5′-ATGAAGGACTTGGCGGATGA-3′ and Reverse: 5′-ATCTCGCCCTTGAGTTTGTCTT-3′; miR-34a: Forward: 5′-CACGGACTCGGGGCATTTGGAGATTTT-3′ and Reverse: 5′-CTGTCTAGATCGCTTATCTTCCCCTTGG-3′; β-actin: Forward, 5′-CTGAGAGGGAAATCGTGCGT-3′ and Reverse, 5′-CCACAGGATTCCATACCCAAGA-3′. U6: Forward, 5′-CTCGCTTCGGCAGCACA-3′ and Reverse, 5′-AACGCTTCACGAATTTGCGT-3′. The relative gene expression was calculated using the 2^−ΔΔ*C*_t_^ method. Experiments were performed in triplicate and repeated three times.

### Luciferase assay

The luciferase assay was performed according to previous reports [23]. Briefly, 5 × 10^4^ cervical cancer cells were grown in a 24-well plate to reach 70% confluence. The wildtype or mutant LDHA 3′UTR containing the predicted miR-34a binding site were ligated into the pmirGLO luciferase reporter vector (Promega, Madison, WI) to generate pmirGLO LDHA-Wt and pmirGLO-LDHA-Mut vectors which were co-transfected with miR-34a precursor or negative control miRNAs by Lipofectamine 2000. Forty-eight hours after transfection, luciferase activity was examined using a Dual-Luciferase Assay Kit (Promega, Madison, WI, U.S.A.). Experiments were performed in triplicate and repeated three times.

### Western blot

Cervical cancer tissue and cells were lysed on ice for 20 min with RIPA lysis buffer (Beyotime, Shanghai, China) plus 1× protease inhibitor cocktail (Sigma, Shanghai, China). Protein concentrations were determined with a BCA Protein Assay Kit (Pierce; Thermo Fisher Scientific). Equal amount of protein from each sample were separated by 10% SDS/PAGE and transferred on to the nitrocellulose membrane. The membrane was first blocked with 5% non-fat milk in PSBT for 1 h at room temperature followed by incubating with primary antibodies at 4°C for overnight. After complete washing by PBST, a horseradish peroxidase (HRP)-conjugated secondary antibody was diluted in PBST buffer for incubating membrane at room temperature for 1 h. After washing by PBST, protein bands from membrane were visualized by an enhanced chemiluminescence detection system (Beyotime, Shanghai, China). β-actin was an internal control. Experiments were repeated three times.

### Statistical analysis

Data were presented as mean ± standard deviation and analyzed by the Prism 6.0 software package (GraphPad Software, Inc.). All experiments were performed for three times independently. The difference between the two groups were analyzed with the Student’s *t* test, one-way ANOVA method was used for analyzing data from multiple groups. The *P*-value <0.05 was considered statistically significant.

## Results

### LncRNA-NEAT1 is positively associated with cervical cancer

**Figure 1:**
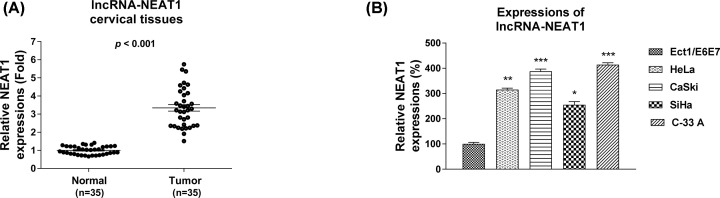
NEAT1 is elevated in cervical cancer tissues and cells (**A**) qRT-PCR was performed to measure the expressions of lncRNA-NEAT1 in cervical cancer tissues and matched normal tissues. (**B**) Expressions of NEAT1 in cervical cancer cells and normal cervical epithelial cells. GAPDH was used as an internal control. All data were shown as mean ± S.D. *, *P*<0.05; **, *P*<0.01; ***, *P*<0.001.

To investigate the roles of lncRNA-NEAT1 in cervical cancer, we initially compared the expressions of NEAT1 in 35 paired human cervical cancer tissues and their adjacent normal tissues by qRT-PCR. Results in Figure 1A showed that the expression of NEAT1 was significantly elevated in cervical cancer tissues compared with that of normal tissues. To further support the up-regulation of NEAT1 in cervical cancer, the expressions of NEAT1 in cervical cancer cell lines, including HeLa, CaSki, SiHa and C33A, as well as a normal cervical epithelial cell line Ect1 were examined. As we expected, the expressions of NEAT1 was significantly up-regulated in cervical cancer cells compared with normal cells. Taken together, the above results revealed that NEAT1 has an oncogenic role in cervical cancer.

### LncRNA-NEAT1 promotes 5-Fu resistance of cervical cancer cells

**Figure 2:**
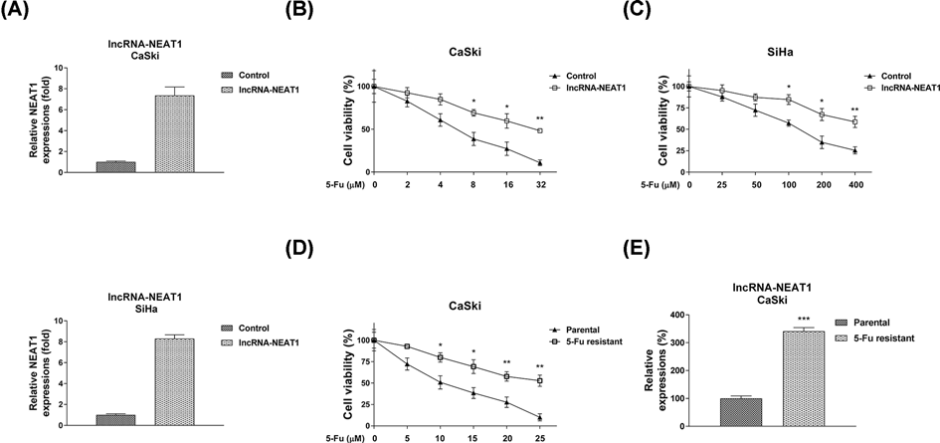
NEAT1 contributes to 5-Fu resistance in cervical cancer cells (**A**) CaSki (upper) and SiHa (lower) cells were transfected with pcDNA-NEAT1 overexpression vector or with the empty vector for 48 h. Expressions of NEAT1 were detected by qRT-PCR. GAPDH was used as an internal control. (**B**) MTT assays were performed to examine the effects of NEAT1 overexpression on the 5-Fu sensitivity of CaSki and (**C**) SiHa cells. (**D**) Establishment of 5-Fu resistant CaSki cells by continually treatments of elevated 5-Fu concentrations. Cells were treated with 5-Fu at 0, 5, 10, 15, 20 or 25 μM for 48 h. The viabilities of resistant and parental cells were measured by MTT assay. (**E**) Expressions of lncRNA-NEAT1 in CaSki parental and 5-Fu resistant cells were detected by qRT-PCR. GAPDH was used as an internal control. All data were shown as mean ± S.D. *, *P*<0.05; **, *P*<0.01; ***, *P*<0.001.

Given the aberrantly oncogenic roles of NEAT1 in cervical cancer, the effect of NEAT1 on the chemoresistance in cervical cancer cells was investigated by gain-of-function studies by transfection of NEAT1 overexpression vector in CaSki and SiHa cells (Figure 2A). Cells were treated with increased concentrations of 5-Fu for 48 h. Consistent results demonstrated exogenous overexpression of NEAT1 rendered cervical cancer cells more resistant to 5-Fu compared with control treatments (Figure 2B,C). To obtain the direct evidence for the NEAT1-promoted 5-Fu resistance, the 5-Fu resistant cell line originating from CaSki cells were established. Cell survival assay demonstrated under 5-Fu treatments, the IC_50_ of 5-Fu resistant cells was 30.31 μM, which is approx. three times of that in CaSki parental cells (Figure 2D). Moreover, we observed the lncRNA-NEAT1 expression was significantly increased in 5-Fu resistant CaSki cells (Figure 2E). Collectively, these results demonstrated that NEAT1 facilitated the 5-Fu resistance of cervical cancer cells.

### LncRNA-NEAT1 down-regulates miR-34a as a sponge in cervical cancer cells

**Figure 3:**
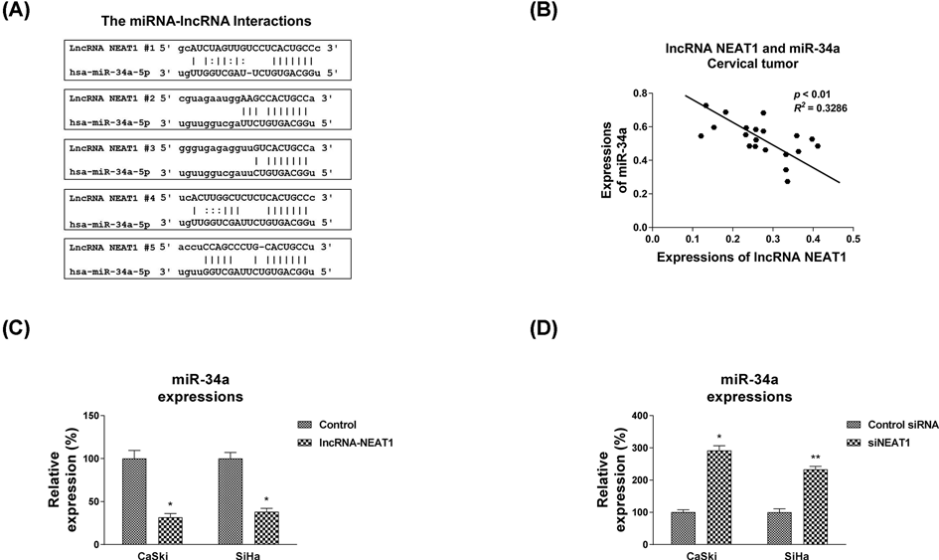
NEAT1 directly binds to miR-34a and negatively regulates miR-34a (**A**) Five binding sites between NEAT1 and miR-34a were analyzed from the bioinformatics database (starbase.sysu.edu.cn). (**B**) The Pearson’s correlation analysis revealed a negative correlation between NEAT1 and miR-34a expressions from cervical cancer tissues. (**C**) NEAT1 overexpression vector or (**D**) NEAT1 shRNA as well as control vector was transfected into CaSki and SiHa cells for 48 h, followed by measurements of miR-34a expressions by qRT-PCR. GAPDH and U6 were used as internal control for lncRNA and miRNAs, respectively. All data were shown as mean ± S.D. *, *P*<0.05; **, *P*<0.01.

The above results demonstrated the association between NEAT1 and cervical cancer cells, the underlying mechanisms of the NEAT1-promoted 5-Fu resistance were further investigated. Increasing evidence revealed that lncRNAs functions as competing endogenous (ce) RNAs through sponging miRNAs [24]. To examine whether NEAT1 promotes the 5-Fu resistance of cervical cancer cells by binding and down-regulating miRNAs as a ceRNA, we search the potential target miRNAs of NEAT1 from Starbase software http://starbase.sysu.edu.cn/. Interestingly, among multiple candidates, seeding region of miR-34a-5p was suggested to bind with NEAT1 at five sites (Figure 3A). Since studies have reported that miR-34a could inhibits the tumorigenesis together with lncRNAs [25,26], we focused on miR-34a as ceRNA of NEAT1. Furthermore, we found a significantly inverse correlation between lncRNA-NEAT1 and miR-34a in cervical tumor specimen (Figure 3B). To validate the interactions between NEAT1 and miR-34a, NEAT1 was overexpressed or inhibited in both CaSki1 and SiHa cells. Expectedly, expressions of miR-34a was significantly attenuated by NEAT1 overexpression compared with control cells (Figure 3C). On the other way, knocking down NEAT1 by shRNA obviously elevated the miR-34a expressions (Figure 3D). These results demonstrated that NEAT1 sponged miR-9-5p and negatively regulated miR-34a expressions in cervical cancer cells.

### miR-34a negatively associates with 5-Fu resistance and inhibits cellular glycolysis rate of cervical cancer cells

**Figure 4:**
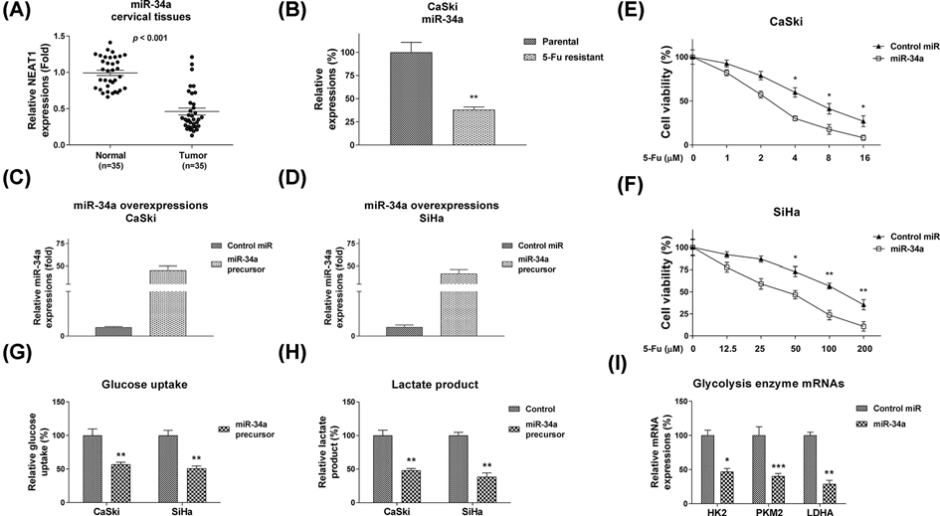
miR-34a is negatively correlated with 5-Fu resistance and inhibits glycolysis (**A**) Comparison of miR-34a levels in cervical tumor tissues and matched normal tissues by qRT-PCR. (**B**) Expressions of miR-34a in CaSki parental and 5-Fu resistant cells detected by qRT-PCR. (**C**) miR-34a or negative control were transfected into CaSki and (**D**) SiHa cells, expressions of miR-34a were measured by qRT-PCR. (**E**) The CaSki and (**F**) SiHa cells without or with miR-34a overexpression were treated with 5-Fu at the indicated concentrations for 48 h, followed by MTT assay to examine the cell viabilities. (**G**) The glucose uptake and (**H**) lactate product were measured in CaSki and SiHa cells without or with miR-34a overexpression. (**I**) The mRNA expressions of glycolysis enzymes, HK2, PKM2 and LDHA were measured by qRT-PCR from CaSki cells without or with miR-34a overexpression. U6 was used as an internal control. All data were shown as mean ± S.D. *, *P*<0.05; **, *P*<0.01; ***, *P*<0.001.

We next assessed whether miR-34a was associated with 5-Fu resistance of cervical cancer. The expression levels of miR-34a in cervical cancer tissues and normal adjacent tissues were compared and consistent results showed miR-34a was down-regulated in cervical tumor specimens (Figure 4A). In addition, miR-34a was significantly lowly expressed in 5-Fu resistant CaSki cells compared with parental cells (Figure 4B), indicating miR-34a has tumor suppressive functions, present it as a new therapeutic target against chemoresistance in cervical cancer. Moreover, direct evidence showed overexpression of miR-34a sensitized CaSki and SiHa cells to 5-Fu (Figure 3E,F). Recent studies revealed dysregulated cellular glycolysis contributed to chemoresistance of cancer cells [20]. To further investigate the underlying mechanisms for the miR-34a-mediated 5-Fu sensitivity, we examined the cellular glycolysis rate of cervical cancer cells without or with miR-34a overexpression. Consistent results showed overexpression of miR-34a apparently suppressed glucose uptake and lactate product, two readouts for detecting cellular glycolysis rates (Figure 3G,H). We also found the glycolysis key enzymes, HK2, PKM2 and LDHA were significantly inhibited by miR-34a (Figure 4I). Taken together, the above results demonstrated a negative correlation between miR-34a and 5-Fu resistance in cervical cancer cells.

### Inhibiting glycolysis sensitizes cervical cancer cells to 5-Fu

**Figure 5:**
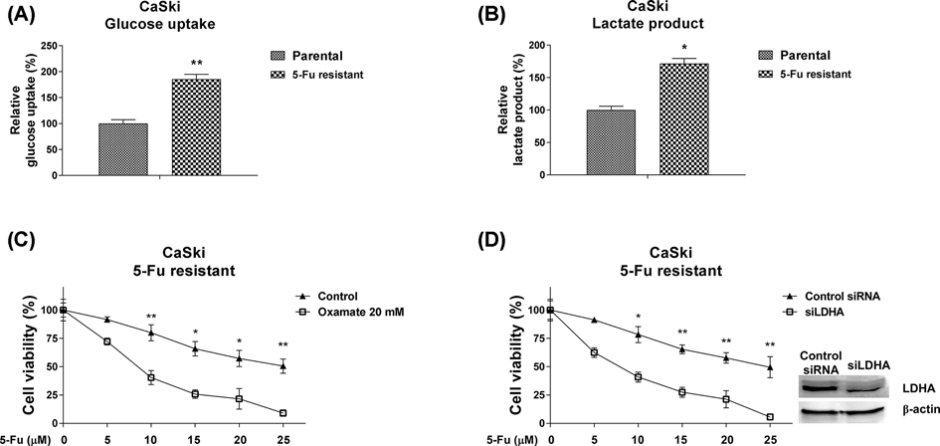
Inhibiting glycolysis sensitizes cervical cancer cells to 5-Fu (**A**) Glucose uptake and (**B**) lactate product from CaSki parental and 5-Fu resistant cells were measured. (**C**) CaSki 5-Fu resistant cells were treated with control PBS and 5-Fu or Oxamate plus 5-Fu at the indicated concentrations for 48 h. The cell viability was measured by MTT assay. (**D**) CaSki 5-Fu resistant cells were transfected with control siRNA or siLDHA for 48 h, followed by 5-Fu treatments at the indicated concentrations for 48 h. The cell viability was measured by MTT assay. All data were shown as mean ± S.D. *, *P*<0.05; **, *P*<0.01.

To evaluate whether the dysregulated glycolysis of cervical cancer cells directly facilitated 5-Fu resistance, the glucose uptake and lactate product were compared in CaSki parental and 5-Fu resistant cells. As we expect, 5-Fu resistant cells displayed obviously elevated glycolysis rates (Figure 5A,B). Further, we observed CaSki 5-Fu resistant cells were sensitized to 5-Fu by either glycolysis inhibitor treatment (Figure 5C) or knocking down LDHA by siRNA (Figure 5D). These data indicating targeting the cellular glycolysis rate could effectively reverse 5-Fu resistance of cervical cancer cells.

### miR-34a sensitizes cervical cancer cells to 5-Fu via directly targeting LDHA

**Figure 6:**
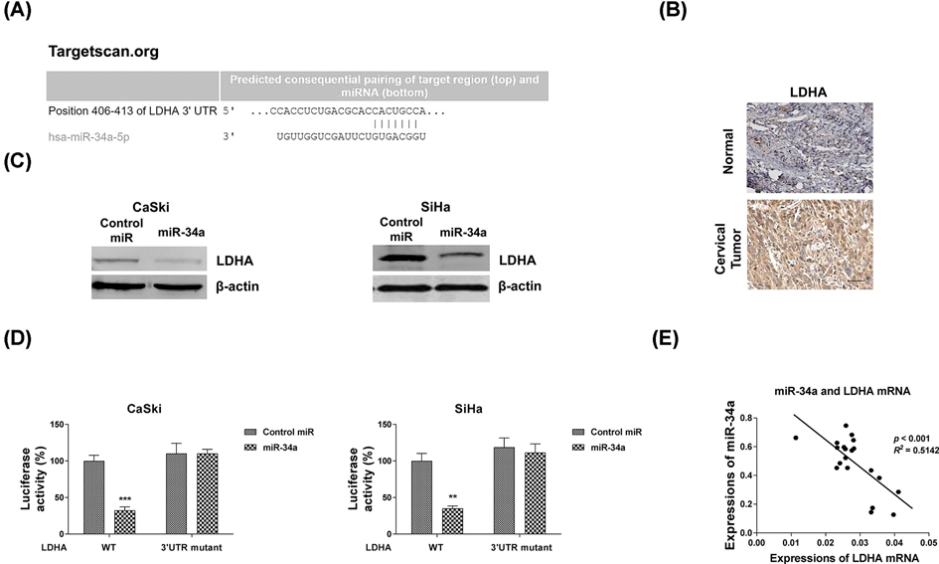
miR-34a directly targets LDHA (**A**) Prediction of the LDHA mRNA-miR-34a binding from TargetScan. (**B**) Immunohistochemistry results of LDHA protein expression from human normal and cervical tumor tissues. (**C**) CaSki (left) and SiHa (right) were transfected with control miRNAs or miR-34a precursor for 48 h, the LDHA protein expressions were examined by Western blot. β-actin was used as a loading control. (**D**) CaSki and SiHa cells were co-transfected with control miRNAs or miR-34a with wildtype or binding site mutant 3′UTR of LDHA in pMIR luciferase reporter vectors. The relative luciferase activities were measured. Experiments were performed in triplicate. (**E**) The negative correlation between miR-34a and LHDA mRNA expressions were observed in cervical cancer tissues. All data were shown as mean ± S.D. **, *P*<0.01; ***, *P*<0.001.

Since miRNAs regulate biological functions through binding to 3′UTR of their targeting mRNAs [16]. To determine the direct targets of miR-34a, we performed mRNA–miRNA binding analysis by bioinformatics prediction from TargetScan. Interestingly, LDHA, a glycolysis key enzyme which catalyzes pyruvate into lactate, was found as a putative downstream effector [19] (Figure 6A). Expectedly, immunohistochemistry experiments showed LDHA protein was significantly up-regulated in human cervical tumor tissues (Figure 6B). Western blot results demonstrated overexpression of miR-34a significantly down-regulated protein expressions of LDHA in two cervical cancer cells (Figure 6C). To identify whether LDHA was the direct target of miR-34a in cervical cancer cells, we constructed luciferase plasmids pGL3-LDHA-WT or pGL3- LDHA-Mut, which contain wildtype or miR-34a binding site mutant 3′UTR of LDHA. They were co-transfected with miR-34a-5p precursor or negative control into CaSki and SiHa cells, respectively. Luciferase assays were performed and the activity of the LDHA WT reporter was obviously blocked, while that of the Mut reporter group did not change (Figure 6D). These results verified that LDHA is a direct target gene of miR-34a-5p in cervical cancer cells. Subsequently, expressions of miR-34a and LDHA mRNAs in cervical cancer tissues displayed a remarkably negative correlation (Figure 6E). The relative higher miR-34a expression tissues were accompanied with lower LDHA mRNAs (Figure 6E). The above results demonstrated miR-34a could directly target LDHA in cervical cancer.

**Figure 7:**
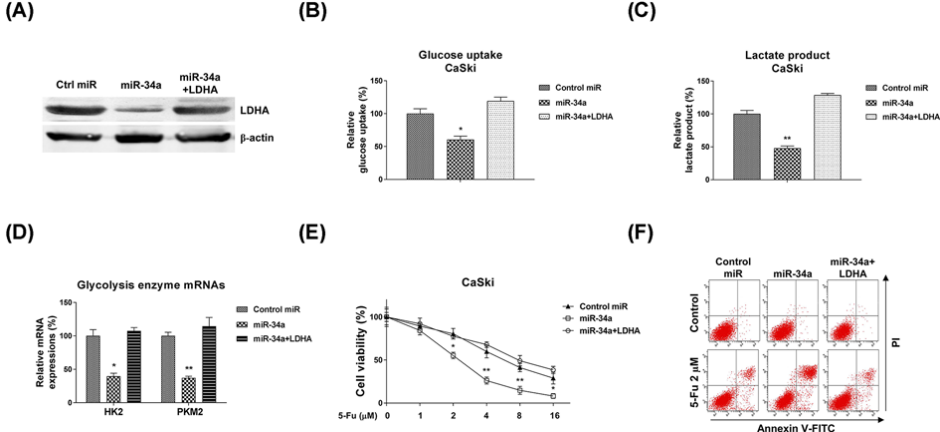
Restoration of LDHA recovered glycolysis and 5-Fu resistance (**A**) CaSki cells were transfected with control miRNAs, miR-34a precursor or miR-34a precursor plus LDHA overexpression vector for 48 h, the LDHA protein expressions were detected by Western blot. β-actin was a loading control. (**B**) The above cells were subjected to glucose uptake and (**C**) lactate product measurements. (**D**) The mRNA levels of glycolysis enzymes were detected by qRT-PCR from the above transfected cells. GAPDH was used as an internal control. (**E**) CaSki cells were transfected with control miRNAs, miR-34a precursor or miR-34a precursor plus LDHA overexpression vector for 48 h, followed by 5-Fu treatment at 0, 1, 2, 4, 8 or 16 µM for 48 h. Cell viabilities were examined by MTT assay. (**F**) The above transfected cells were treated with 5-Fu at 2 µM for 48 hours, the cell death was examined by Annexin V/FITC assay. Experiments were performed in triplicate. All data were shown as mean ± S.D. *, *P*<0.05; **, *P*<0.01.

To elucidate whether the miR-34a-regulated 5-Fu sensitivity and glycolysis inhibition via targeting LDHA, we performed rescue experiments by transfection of CaSki cells with control miRNAs, miR-34a along or miR-34a plus LDHA overexpression vector. Western blot results indicated co-transfection of miR-34a and LDHA successfully restored the LDHA protein expression (Figure 7A). Expectedly, CaSki cells with LDHA restoration showed recovery of glucose uptake and lactate product (Figure 7B,C). Moreover, glycolysis key enzymes, HK2 and PKM2 were significantly recovered to normal expressions in miR-34a and LDHA co-transfected cells (Figure 7D). We assessed whether rescue of LDHA in miR-34a overexpressed cells could overcome the 5-Fu sensitivity. Subsequently, cell viability assay and Annexin V/PITC apoptosis assay consistently demonstrated that CaSki cells with co-transfection of miR-34a and LDHA were re-sensitized to 5-Fu (Figure 7E,F). Moreover, the rescue phenotypes were observed in another cervical cancer cell line, SiHa (Supplementary Figure S1A–C). Taken together, the rescue results verified the miR-34a-suppressed glycolysis and 5-Fu resistance were through directly targeting LDHA in cervical cancer cells.

### Inhibition of NEAT1 sensitizes cervical cancer cells to 5-Fu through promoting miR-34a/LDHA axis

**Figure 8:**
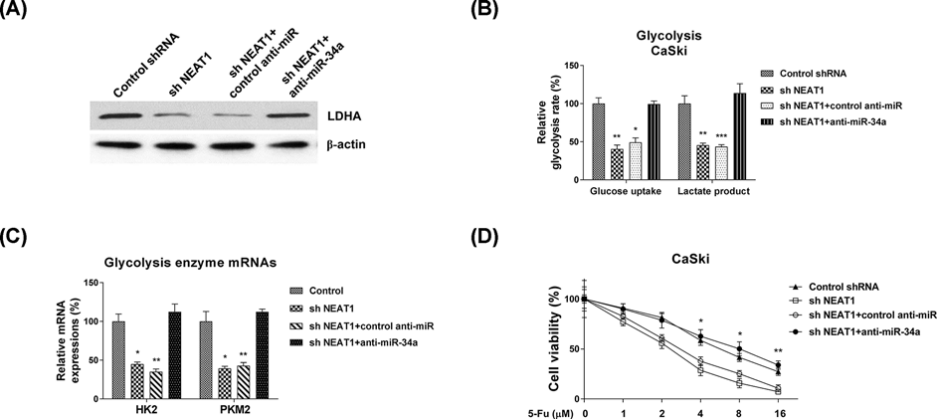
The NEAT1-promoted 5-Fu resistance is through miR-34a/LDHA axis (**A**) CaSki cells were transfected with control shRNA, NEAT1 shRNA alone, NEAT1 shRNA plus control antisense, or NEAT1 shRNA plus miR-34a antisense for 48 h, the LDHA protein expressions were detected by Western blot. β-actin was a loading control. (**B**) The above cells were subjected to glucose uptake and lactate product measurements. (**C**) The mRNA levels of glycolysis enzymes were detected by qRT-PCR from the above transfected cells. GAPDH was used as an internal control. (**D**) The above transfected CaSki cells were treated by 5-Fu at 0, 1, 2, 4, 8 or 16 uM for 48 h. Cell viabilities were examined by MTT assay. Experiments were performed in triplicate. All data were shown as mean ± S.D. *, *P*<0.05; **, *P*<0.01; ***, *P*<0.001.

We further characterized whether the NEAT1-promoted 5-Fu resistance was dependent on miR-34a-LDHA axis. CaSki cells were transfected with control shRNA, NEAT1 shRNA, NEAT1 shRNA plus control miRNAs antisense or NEAT1 shRNA plus miR-34a antisense. As shown in Figure 8A, Supplementary Figure S2, knocking down NEAT1 significantly down-regulated LDHA and up-regulated miR-34a. However, such regulations were further reversed by miR-34a inhibition (Figure 8A, Supplementary Figure S2), indicating the NEAT1-promoted LDHA expression was through miR-34a inhibition. Consistently, co-transfection of NEAT1 shRNA and miR-34a inhibitor apparently recovered cellular glycolysis rate (Figure 8B) and enzyme expressions (Figure 8C). Moreover, CaSki cells with co-transfection of NEAT1 shRNA and miR-34a inhibitor showed increased 5-Fu resistance, compared with NEAT1 knockdown alone (Figure 8D). In summary, the above results elucidated that NEAT1 suppressed miR-34a to promote the glycolysis and 5-Fu resistance in cervical cancer, presenting the NEAT1/miR-34a-5p/LDHA axis as an effective target on regulating 5-Fu sensitivity of cervical cancer cells.

## Discussion

Cervical cancer is one of the most common malignancy and the fourth prominent cancer-caused mortality in women [1–4]. Although the cervical cancer patients who received surgical operations, radiotherapy and chemotherapy demonstrated certain anti-cancer effects, prognosis of cervical cancer is still unsatisfied due to the acquired chemoresistance [4]. Therefore, understanding the molecular mechanisms underlying the chemoresistance of cervical cancer emerges as an urgent task. In the present study, we characterized the 5-Fu resistance cervical cancer cells and found lncRNA-NEAT1 was positively associated with 5-Fu resistance. Moreover, the cellular glycolysis rates were significantly increased in 5-Fu resistant cervical cancer cells, suggesting targeting the NEAT1-mediated glycolysis could effectively overcome chemoresistance.

Accumulating evidence have demonstrated that NEAT1 functions as an oncogene in diverse cancers [11–15,27–29]. For instance, lncRNA NEAT1 promotes cell proliferation and migration of gastric cancer, cervical cancer, endometrial cancer and bladder cancer [15,27,28,29]. In breast cancer, NEAT is positively correlated with poor survival rate of patients and contributes to paclitaxel resistance in ovarian cancer cells through miR-194/ZEB1 axis [13]. Currently, the underlying mechanisms of NEAT1 in regulating 5-Fu resistance of cervical cancer have not been elucidated. We found NEAT1 was significantly up-regulated in cervical cancer patients and cells, consistent with previous report [27]. Furthermore, by establishing 5-Fu resistant cervical cancer cell line, we observed obviously up-regulated NEAT1 in 5-Fu resistant cells compared with parental cells, suggesting NEAT1 is positively associated with 5-Fu resistance of cervical cancer.

It has been acknowledged that lncRNAs and miRNAs take charge of diverse processes of tumors [8,9,16,17]. Interestingly, recent studies have demonstrated that lncRNA could interact with microRNAs as competing endogenous RNAs to suppress miRNAs expression [24,30]. Our findings firstly illustrated NEAT1 functions as ceRNAs to sponge miR-34a, thereby modulating the de-repression of LDHA, the direct target of miR-34a. We showed consistent results that overexpression of NEAT1 inhibited miR-34a expressions. On the other hand, NEAT1 down-regulation led to up-regulated expression of miR-34a. Importantly, such invert correlation between NEAT1 and miR-34a was further verified in cervical cancer tissues. We identified LDHA as a direct target of miR-34a by luciferase assay. Furthermore, restoration of LDHA in miR-34a overexpressing cells successfully rescued the glycolysis rate, suggesting the miR-34a-mediated glycolysis inhibition was through targeting LDHA.

Proliferating tumor cells display increased lactate production during glucose metabolism even in the presence of sufficient oxygen [19]. This phenomenon is known as the Warburg effect, which is recognized as new marker of cancer cells. Moreover, the Warburg effect is tightly related to tumor proliferation, progression and drug resistance [20,31,32]. In this study, we focused our attention on the effects of the lncRNA-NEAT1-promoted glycolysis on 5-Fu resistance. In accordance with our expectation, overexpression of NEAT1 effectively de-sensitized cervical cancer cells through glycolysis up-regulation. In addition, glycolysis inhibition by either glycolysis inhibitor or LDHA knockdown led to increased 5-Fu sensitivity of cervical cancer cells. Finally, we examined whether the NEAT1-promoted 5-Fu resistance was through miR34a/LDHA pathway. In NEAT1 knockdown cells, the cervical cancer cells showed increased 5-Fu sensitivity. Expectedly, co-transfection of NEAT1 shRNA and miR-34a inhibitor in cervical cancer cells showed recovered glycolysis and 5-Fu resistance, suggesting that NEAT1 suppressed miR-34a to promote the glycolysis and 5-Fu resistance in cervical cancer.

In summary, we propose that the negative association between NEAT1 and miR-34a in cervical cancer cells contributes to the dysregulated cellular glycolysis, resulting in 5-Fu resistance. Although direct targeting LDHA by miR-34a has been revealed in other cancers, in speaking of lncRNA NEAT1, its function as a ceRNA of miR-34a to further de-suppress LDHA in cervical cancer has not been elucidated. Our future studies will aim to investigate the above molecular pathway in an *in vivo* animal model. Our findings suggest that targeting the NEAT1-mediated miR-34a/LDHA-glycolysis axis might be regarded as a promising therapeutic strategy against chemoresistant cervical cancer.

## Supplementary Material

Supplementary Figures S1-S2

## Data Availability

The datasets used and/or analyzed during the current study are available from the corresponding author on reasonable request.

## Abbreviations

ceRNA: competitive endogenous RNA
LDHA: Lactate Dehydrogenase-A
lncRNA: long noncoding RNA
MTT: 3-(4,5-dimethylthiazol-2-yl)-2,5-diphenyltetrazolium bromide
NEAT1: Nuclear Enriched Abundant Transcript 1
5-Fu: 5-fluorouracil
