# Supplementary material for: Inhibition of lncRNA-NEAT1 sensitizes 5-Fu resistant cervical cancer cells through de-repressing the microRNA-34a/LDHA axis
Source: Biosci Rep. 2021 Jul 22;41(7):BSR20200533. doi: 10.1042/BSR20200533 (PMC8298262; doi:10.1042/BSR20200533)
Supplement: Supplementary Figures S1-S2 [file BSR-2020-0533_supp.pdf]

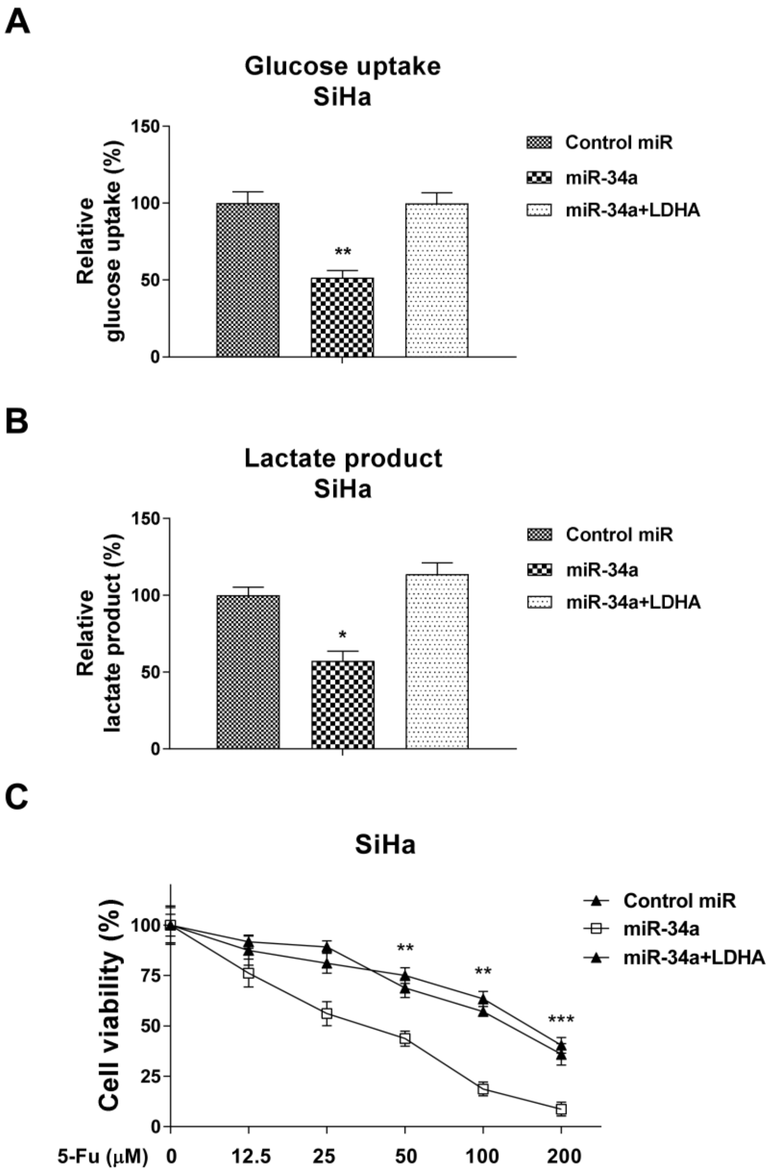

Figure S1. Rescue experiments for miR-34a-LDHA axis from SiHa cells. (A) SiHa cells were transfected with control miRNAs, miR-34a precursor or miR-34a precursor plus LDHA overexpression vector for 48 hours, the glucose uptake and (B) lactate product were examined. (C) The above transfected SiHa cells were treated with 5-Fu at the indicated concentrations for 48 hours. Cell viabilities were examined by MTT assay. All data were shown as mean ± S.D. *, $p<0.05$; **, $p<0.01$; ***, $p<0.001$.

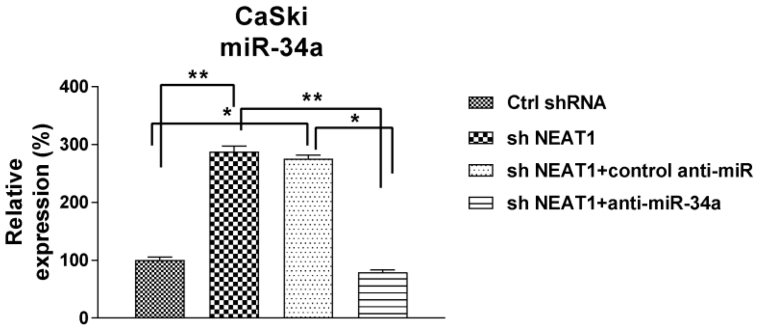

Figure S2. Expressions of miR-34a were determined by qRT-PCR in CaSki cells transfected with control shRNA, NEAT1 shRNA alone, NEAT1 shRNA plus control antisense, or NEAT1 shRNA plus miR34a antisense. All data were shown as mean ± S.D. *, *p*<0.05; **, *p*<0.01.

Supplementary material 1. Original WB results for figure 5D, 6C, 7A, 8A
